# Evaluation of the effect of GM-CSF blocking on the phenotype and function of human monocytes

**DOI:** 10.1038/s41598-020-58131-2

**Published:** 2020-01-31

**Authors:** Noushin Lotfi, Guang-Xian Zhang, Nafiseh Esmaeil, Abdolmohamad Rostami

**Affiliations:** 1 Department of Neurology, Thomas Jefferson University, Philadelphia, PA USA; 2 Department of Immunology, School of Medicine, Isfahan University of Medical Sciences, Isfahan, Iran

**Keywords:** Cell migration, Translational research

## Abstract

Granulocyte-macrophage colony-stimulating factor (GM-CSF) is a multipotent cytokine that prompts the proliferation of bone marrow-derived macrophages and granulocytes. In addition to its effects as a growth factor, GM-CSF plays an important role in chronic inflammatory autoimmune diseases such as multiple sclerosis and rheumatoid arthritis. Reports have identified monocytes as the primary target of GM-CSF; however, its effect on monocyte activation has been under-estimated. Here, using flow cytometry and ELISA we show that GM-CSF induces an inflammatory profile in human monocytes, which includes an upregulated expression of HLA-DR and CD86 molecules and increased production of TNF-α and IL-1β. Conversely, blockage of endogenous GM-CSF with antibody treatment not only inhibited the inflammatory profile of these cells, but also induced an immunomodulatory one, as shown by increased IL-10 production by monocytes. Further analysis with qPCR, flow cytometry and ELISA experiments revealed that GM-CSF blockage in monocytes stimulated production of the chemokine CXCL-11, which suppressed T cell proliferation. Blockade of CXCL-11 abrogated anti-GM-CSF treatment and induced inflammatory monocytes. Our findings show that anti-GM-CSF treatment induces modulatory monocytes that act in a CXCL-11-dependent manner, a mechanism that can be used in the development of novel approaches to treat chronic inflammatory autoimmune diseases.

## Introduction

Granulocyte-macrophage colony-stimulating factor (GM-CSF) is a multipotent cytokine that stimulates the proliferation of bone marrow-derived macrophages and granulocytes. Various cell types produce this cytokine, including activated T cells, monocytes/macrophages, B cells, NK cells, endothelial, epithelial, and fibroblasts cells^1^. GM-CSF has been identified as a major cytokine in chronic inflammatory autoimmune diseases such as multiple sclerosis (MS) and rheumatoid arthritis (RA)^2,3^ GM-CSF plays a crucial role in RA progression and augments inflammatory immune responses in synovia^4,5^. Moreover, GM-CSF-producing CD4^+^ T cells in the blood and lesions of untreated MS patients correlate with disease severity^6^.

We have shown that GM-CSF is necessary for the pathogenicity of Th17 cells in experimental autoimmune encephalomyelitis, the prototypical animal model for MS^7^. GM-CSF exerts its function by binding to its receptor, which is composed of two different subunit α (CD116; GM-CSF Rα) and β chains (CD131; GM-CSF Rβ) with low and high affinity, respectively. The alpha subunit is involved in ligand-specific binding while the beta chain plays a central role in the signal transduction pathway^8^. GM-CSF signaling affects the survival and activation of myeloid cells, dendritic cell (DC) differentiation and M1 macrophage phenotype polarization; it boosts antigen presentation, induces phagocytosis, recruits monocytes and other myeloid populations from bone marrow to circulation and promotes chemotaxis^9,10^.

It has been recently demonstrated that CCR2^+^Ly6C^hi^ inflammatory monocytes are a target of GM-CSF in CNS autoimmunity by stimulating inflammatory monocytes and their conversion into pathogenic macrophage-derived dendritic cells^11–13^. GM-CSF-activated monocytes migrate across the blood-brain barrier (BBB) and mediate BBB rupture by increasing expression of the endothelial adhesion molecules ICAM-1 and VCAM-1^14,15^. GM-CSF also induces CCR2 expression in monocytes, which gives them an increased ability to cross the BBB. In EAE and MS, the CCR2-CCL2 axis has been previously shown to be a significant driver of inflammatory leukocyte infiltration into the CNS, and its activity positively correlates with disease pathogenesis^16–18^. Migration of leukocytes into the CNS is also mediated by CXCL9 and CXCL10 produced by glial cells^19^. Activated T lymphocytes in MS patients express CXCR3, which is the corresponding receptor of CXCL9, CXCL10, and CXCL-11 chemokines^20^.

It has been previously shown that while CXCL9 is a homing chemokine in the CNS, CXCL10, and CXCL-11 are induced after inflammation, and their role in inflammation is less clear^21–23^.

CXCL10 is involved in intrathecal inflammation^24^. Interestingly, CXCL-11 is upregulated in MS patients after IFN-β therapy and the decrease in the number of relapses may be linked to the increase in CXCR3 ligands in the serum of IFN-β-treated MS patients^25^.

In this study, we analyzed the effect of GM-CSF on the phenotype and function of human monocytes. We found that GM-CSF treatment induces an inflammatory phenotype in monocytes, while endogenous GM-CSF blocking is accompanied by an immunomodulatory phenotype. Further, GM-CSF blockade promoted CXCL-11 expression, and recombinant CXCL-11 inhibited the GM-CSF-induced proinflammatory impact of monocytes on T cells. Our findings show that one of the mechanisms by which GM-CSF induces inflammatory monocytes is the inhibition of CXCL-11 production and that this chemokine may be harnessed to suppress deleterious inflammatory responses observed in chronic inflammatory diseases such as MS.

## Methods

### Isolation of human monocytes and culture treatments

All subjects gave informed consent before their participation in the current study. All human studies were approved by the Institutional Review Board (IRB) of Thomas Jefferson University, and all methods were performed in accordance with the relevant guidelines and regulations. Whole blood samples were collected from healthy donors and peripheral blood mononuclear cells (PBMCs) were enriched by gradient centrifugation in Ficoll. CD14^+^ monocytes were isolated by positive selection using magnetic beads following the manufacturer’s instructions (Miltenyi Biotec, Bergisch Gladbach, Germany). The purity of cells was above 90%, measured by flow cytometry. Monocytes were seeded (1 × 10^6^/ml) in 24-well plates and cultured in Iscove’s Modified Dulbecco’s Medium (IMDM) (Gibco, Gaithersburg, MD, USA) supplemented with 10% FBS, 1% penicillin/streptomycin antibiotic (Gibco), 2 mM glutamine and 2β- Mercaptoethanol (50 µg/ml, Gibco). Monocytes were activated with lipopolysaccharide (100 ng/mL, Sigma-Aldrich) for 18 h at 37 °C in the presence of recombinant human GM-CSF (10 ng/mL, R&D Systems, Minneapolis, MN, USA) or anti-GM-CSF (10 µg/mL, Biolegend, San Diego, CA). LPS-activated cells (mature monocytes) cultured with PBS were used as controls and culture of monocytes without LPS stimulation were considered as immature cells.

### RNA extraction, cDNA synthesis, and qPCR array

RNA was extracted using the RNeasy Mini Kit (Qiagen, Hilden, Germany), and the RNA concentration and quality were determined with Nanodrop (Thermofisher Scientific, Waltham, MA, USA). cDNA synthesis was performed from 1 µg of RNA using High-Capacity cDNA Reverse Transcription Kit (Applied Biosystems, Foster City, CA, USA) according to the manufacturer’s instructions. Real-time PCR was performed using the TaqMan™ Array Human Immune Response (Applied Biosystems). Real-time PCR for CXCL-11 (Hs03003631_g1) was conducted according to the manufacturer’s instructions using TaqMan reagents (ThermoFisher). Relative expression was calculated following the 2^-ΔΔCT^ method, where 18 s (Hs03003631_g1) was considered the housekeeping gene.

### Flow cytometric analysis

For assessment of surface and intracellular cytokine expression, monocytes were collected after 24 hours and stimulated for three hours with 50 ng/ml PMA (Sigma-Aldrich, St. Louis, MO,USA), 500 ng/ml ionomycin (Sigma-Aldrich), and 1 µg/ml GolgiPlug (BD Biosciences, San Jose, CA, USA). Cells were stained with anti-CD14 (M5E2, Biolegend), anti-CD16 (3G8, Biolegend.), anti-CD11b (ICRF44, Biolegend), anti-HLA-DR (L243, Biolegend), anti-CD80 (2D10, Biolegend), anti-CD86 (IT2.2, Biolegend), anti-CD83 (HB15e, Biolegend) and anti-PDL1(29E.2A3 Biolegend). Surface staining was performed for 20 min at 4 °C in the dark, and after washing cells were fixed using 100 µl/tube fixation buffer at room temperature for 30 min (Thermofisher Scientific). Subsequently, the monocytes were permeabilized with 100 µl/tube Permeabilization Buffer (Thermofisher Scientific) and then stained with fluorochrome-conjugated antibodies for intracellular markers including anti-IL-10 (JES3-9D7, Biolegend), anti-TNF-α (MAb11, Biolegend) and anti-IL-1β (H1b-98, Biolegend), anti-IL-27 (B032F6, Biolegend) overnight at 4 °C in the dark.

Also, the cells harvested from T- cells × monocytes co-culture treatments were stained for surface and intracellular markers with fluorochrome-conjugated antibodies including anti-CD3(SK7, Thermofisher Scientific), anti-CD4 (OKT4, Biolegend) anti-PDL-1 (29E.2A3, Biolegend), anti-IL-10 (JES3-19F1, Biolegend), anti-IFN-γ (B27, Biolegend), anti-RORɤt (Q21-559, BD Biosciense), anti-CD39 (A1, Biolegend).

Samples were acquired on a BD FACS Aria Fusion (BD Biosciences) flow cytometry instrument and data analyzed using Flowjo software 10. The instrument calibration was examined before running the samples with BD FACSDiva™ CS&T research beads (CS&T research beads, BD Biosciences).

### Co-culture experiments

To examine the effects of GM-CSF, CXCL11 and their blockade on T cell responses, human monocytes and naïve T cells were isolated from peripheral blood of healthy volunteers by magnetic cell isolation according to the company’s instructions (Miltenyi Biotec). Purified monocytes at 2 × 10^4^ were seeded to U bottom 96 well plates and cultured in IMDM culture medium, which contained 10% FBS and 1% penicillin-streptomycin, in 7 groups with different treatment conditions including PBS, GM-CSF (10 ng/ml), Anti-GM-CSF (10 µg/ml), RHCXCL11(15 ng/ml) (R&D Systems), RHCXCL11 + GM-CSF, Anti CXCL11 (10 µg/ml) (R&D Systems, USA), and Anti-GM-CSF + Anti-CXCL11 for 24 hours. The following day, isolated naïve T cells were labeled with Cell Trace Violet (Thermo Fisher) following the manufacturer’s instructions. T cells were then stimulated with the soluble anti-CD3 antibody at a concentration of 1 µg/ml. T cells were then added (1 × 10^5^ cells per well) to the same monocyte wells for cell-cell interactions and kept humidified in a 5% CO2 incubator at 37 °C for 72 hours. After that time, cell culture supernatant was carefully removed and immediately frozen at −20 °C. To measure the proliferative capacity of T cells, CFSE intensity in the cells was assessed by flow cytometry.

### Enzyme-linked immunosorbent assay (ELISA)

Supernatants from cultures described above were stored at −20°C until used to detect CXCL-11, IL-10, IL-1β, TNF-α, IL-27, and IFN-γ by ELISA kits following the manufacturer’s recommendations (R&D Systems).

### Statistical analysis

Statistical analyses were done using Graph Pad Prism version 7. Comparison between groups was performed using Student’s t-test (two groups) and one-way ANOVA tests (three or more groups). Data are shown as mean ± SEM. P values lower than 0.05 were considered statistically significant.

## Results

### GM-CSF induces an inflammatory profile in monocytes

**Figure 1:**
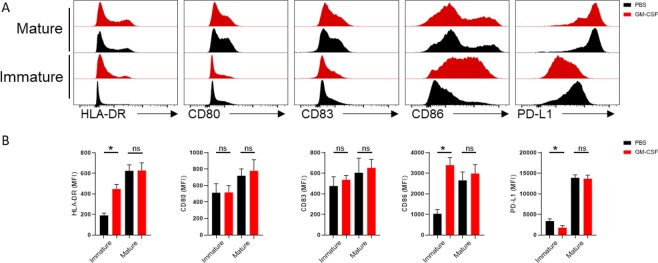
Overexpression of MHCII and costimulatory molecules in GM-CSF treated human monocytes. (**A**) Graphs summarize flow cytometry findings. (**B**) Results are reported as mean ± SEM from three independent experiments.

To examine the effect of GM-CSF on monocyte maturation, we stimulated freshly isolated monocytes with LPS in the presence or absence of GM-CSF for 18 h and analyzed the expression of molecules associated with monocyte maturation and antigen presentation. We found that GM-CSF induced a significant increase in the expression of MHC-II and CD86 compared to the PBS-treated groups. Inversely, the expression level of PDL-1 was decreased after GM-CSF treatment. However, no significant differences were found in CD80, CD83 expression between treatment and control groups (Fig. 1A,B)

**Figure 2:**
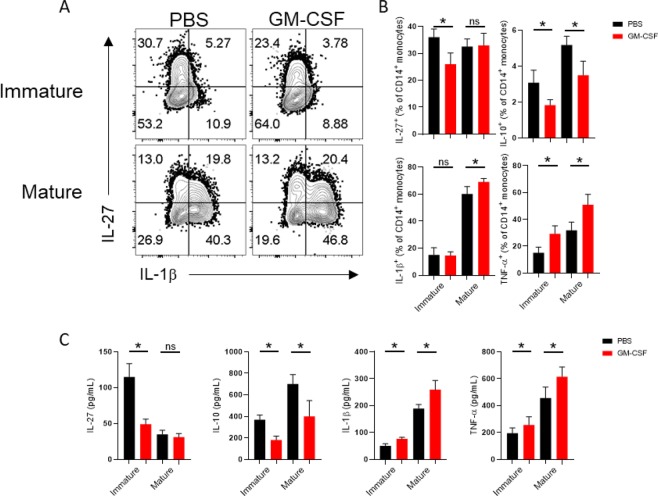
GM-CSF treatment induces an inflammatory phenotype in monocytes. Representative flow cytometry dot plots show the level of IL-27 and IL-1β. (**A**) Graphs indicate the percentage of cells producing IL-27, IL-10, and IL-1β and TNF-α in the CD14^+^ gate. (**B**) Cytokine levels in culture supernatant were verified by ELISA (**C**). Results are representative of three independent experiments and are expressed as mean ± SEM.

Given that HLA-DR and CD86 are necessary first and second signals in antigen-presentation^26^, we investigated whether cytokines, which are the third signal, would be affected by GM-CSF treatment. We found that GM-CSF significantly decreased the level of anti- inflammatory cytokines like IL-27 and Il-10 (Fig. 2A,B). On the other hand we observed the increased levels of TNF-α and IL-1β in GM-CSF treated compared with PBS-treated cultures (Fig. 2A–C). The level of IL-1β and TNF- α was found to be significantly increased in culture supernatants as detected by ELISA (Fig. 2C). Of note, IL-10 and IL-27 was significantly reduced after GM-CSF treatment (Fig. 2C).

### Blockade of endogenous GM-CSF induces an immunomodulatory phenotype in monocytes

Our results showed that GM-CSF induced a pro-inflammatory profile in monocytes, an effect that has been previously described in the mouse system^27^.

**Figure 3:**
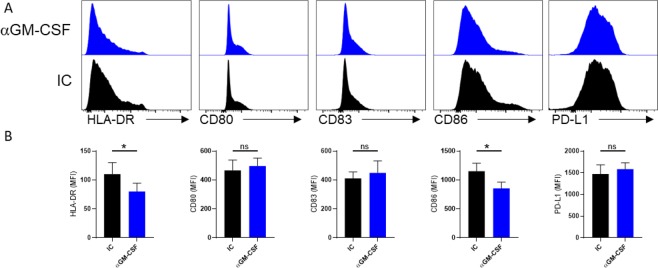
GM-CSF blockade decreases the expression of MHCII and co-stimulatory molecules in monocytes. (**A**) Representative histograms show the isotype controls (black) and the expression level of inflammatory and suppressive markers after anti-GM-CSF antibody treatment (blue). (**B**) Graphs summarize flow cytometry findings. Results are presented as mean ± SEM from three independent experiments.

Given that monocytes also produce small amounts of GM-CSF^28^, we wanted to test if blockage of endogenous GM-CSF would affect the maturation of monocytes after exposure to LPS. To block endogenous GM-CSF, LPS-activated monocytes were cultured in the presence of anti-GM-CSF monoclonal antibodies. Our results showed that anti-GM-CSF treatment of monocytes significantly reduced expression of HLA-DR and CD86 compared to the control group (Fig. 3A,B).

Interestingly, PD-L1 expression remained unaltered (Fig. 3A,B). PD-L1 induced modulation or anergy of PD1^+^ cells^29^. The fact that GM-CSF supplementation or blockage had little or no effect on PD-L1 expression suggests that GM-CSF plays no role in the PD-L1-PD1 signaling axis.

**Figure 4:**
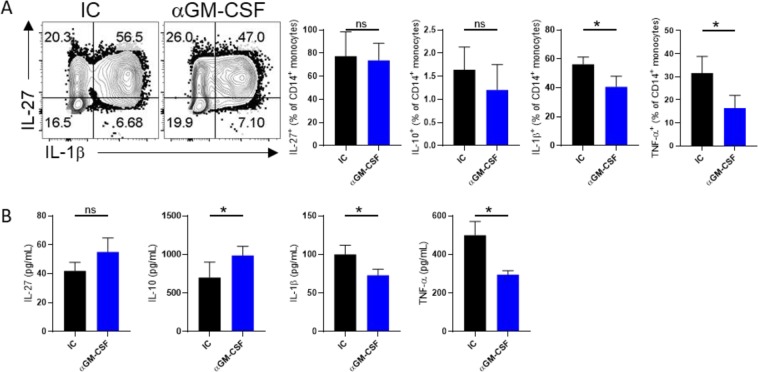
GM-CSF blockade induces an immunomodulatory phenotype of monocytes. Representative flow cytometry dot plots **(A)** Graphs show the percentage of cells producing IL-27, IL-10, IL-1β and TNF-a in the CD14^+^ gate after GM-CSF blockage (**A**). Cytokine production was measured by ELISA. (**B**) Results are presented as mean ± SEM from three independent experiments.

We also found that anti-GM-CSF treatment decreased the expression of TNF-α, IL-1β compared with controls (Fig. 4A,B). Conversely, anti-GM-CSF-treated monocytes significantly produced more IL-10 than controls while, there was no significant difference in IL-27 level between groups (Fig. 4A,B). Collectively, our data show that GM-CSF induced pro-inflammatory phenotype in monocytes and its blockage induced anti-inflammatory cells.

### CXCL-11 is suppressed by GM-CSF

**Figure 5:**
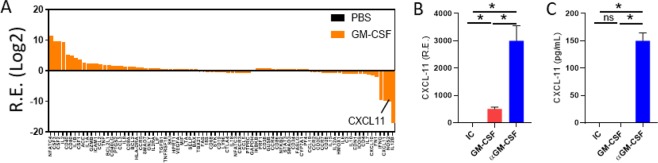
GM-CSF blockade promotes CXCL-11 expression. QPCR array results showed the suppression of CXCL-11 expression by GM-CSF (**A**). CXCL-11 expression was assessed using quantitative real-time PCR (**B**). The production level of CXCL-11 was analyzed by ELISA (**C**). Results are presented as mean ± SEM from three independent experiments.

Our results confirmed that GM-CSF induces the expression of antigen-presenting molecules and stimulates the production of inflammatory cytokines by monocytes. We also showed that blockage of endogenous GM-CSF had the opposite effect by inducing anti-inflammatory monocytes. We then sought to investigate which genes are negatively regulated by GM-CSF. We extracted RNA from monocytes treated with GM-CSF. Monocytes treated with PBS were used as controls. The RNA was reverse transcribed to cDNA, and the cDNA was run in a PCR Array plate that analyzed 96 genes. Our results showed that GM-CSF altered the total gene expression levels in human monocytes (Fig. 5A). We found that CXCL-11 was among the genes least expressed in GM-CSF-treated monocytes (Fig. 5A). Conversely, we confirmed that anti-GM-CSF treatment upregulated CXCL-11 at the gene and protein levels (Fig. 5B,C, respectively).

**Figure 6:**
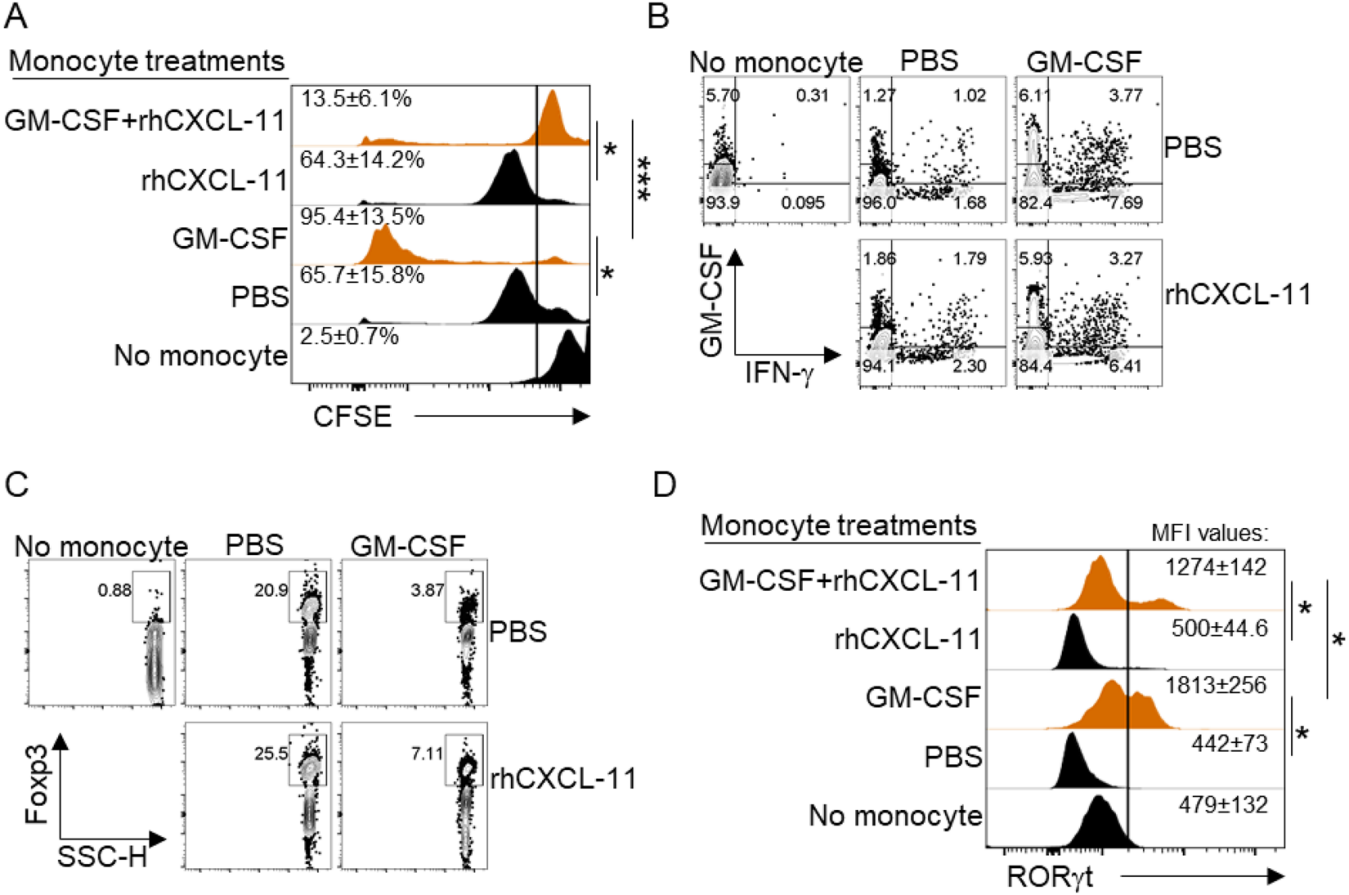
CXCL-11 inhibits GM-CSF-induced pro-inflammatory effects of monocytes on CD4^+^ T cells. CFSE histograms show the rate of proliferation of T cells under various treatments. (**A**) Representative flow cytometry dot plots of IFN-g and GM-CSF with different treatments. (**B**) Flow cytometry Dot plots of FOXP3 expression with distinct treatments (**C**). Histogram analyses show the expression level of RORɤt (**D**). Results are presented as mean ± SEM from three independent experiments.

CXCL-11 is involved in the generation of regulatory T cells and in the suppression of inflammation^30^. To investigate the contribution of CXCL-11 as a modulatory chemokine of anti-GM-CSF-treated monocytes, we performed a co-culture experiment between syngeneic monocytes and naive T cells. Monocytes were treated with GM-CSF and LPS or with LPS only. To assess their ability to stimulate T cell proliferation, GM-CSF-treated monocytes were cultured with Cell Trace Violet (CTV)-labeled naïve CD4^+^ T cells in the presence or absence of recombinant human CXCL-11. After the incubation period, cells were collected, and the dye decay was measured by flow cytometry. Our data showed that while GM-CSF-treated monocytes stimulated a high proliferative response from T cells, the addition of rhCXCL-11 significantly suppressed this response (Fig. 6A). We also analyzed the phenotype of T cells that were cultured with monocytes and found that IFN-γ production was significantly suppressed in cultures conducted in the presence of rhCXCL-11 (Fig. 6B). Interestingly, the expression of FOXP3 in T cells, which is related to suppressive activity of Treg cells was increased in existence of the CXCL11 (Fig. 6C). Moreover, the Th17-related transcriptional factor RORγt was considerably decreased, in T cells cultured in the presence of rhCXCL-11 (Fig. 6D). These results demonstrate that CXCL-11 is an inhibitor of T cell activation and can overcome the inflammatory effect that GM-CSF exerts on monocytes.

### CXCL-11 is a significant regulatory chemokine produced by anti-GM-CSF-treated monocytes

**Figure 7:**
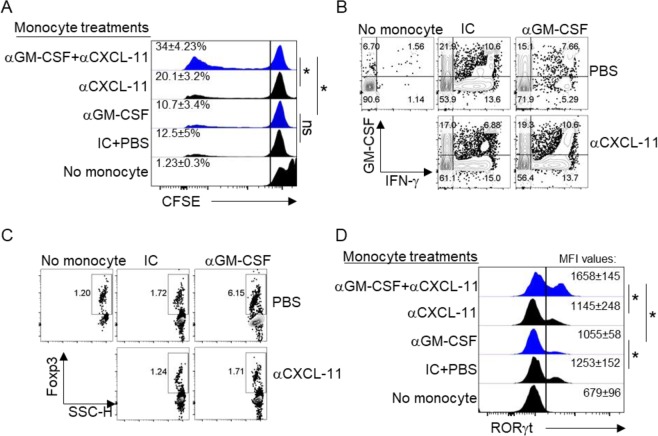
CXCL-11 is required for the immunomodulatory effects of monocytes after GM-CSF blockade. CFSE histograms show the proliferation rate of T cells after blockade of rhCXCL-11 and GM-CSF (**A**). Representative flow cytometry dot plots for IFN-γ after blockage of CXCL-11 and GM-CSF (**B**). Flow cytometry Dot plots of FOXP3 expression with distinct treatments (**C**). Histogram analysis shows the expression level of RORɤt in different treatment conditions (**D**). Results are presented as mean ± SEM from three independent experiments.

We found that GM-CSF directly suppresses the production of CXCL-11 while anti-GM-CSF treatment had the opposite effect (Fig. 5). We then showed that addition of rhCXCL-11 to co-cultures of GM-CSF-treated monocytes overcame the inflammatory phenotype of monocytes and modulated T cells towards an immunoregulatory phenotype (Fig. 6). We then investigated whether blockage of CXCL-11 would hamper the beneficial effect of anti-GM-CSF treatment on monocytes. We performed a co-culture experiment with anti-GM-CSF-treated monocytes and CTV-labeled T cells obtained from the same donors. Culture conditions included cells cultured in the presence or absence of blocking antibodies to CXCL-11. Our results showed that while anti-GM-CSF-treated monocytes had little impact on the proliferation of T cells, blockage of CXCL-11 rescued their proliferation (Fig. 7A). Also, we figured out that blockage of CXCL-11 would result in increase in the production of IFN- γ (Fig. 7B). Moreover, we found that CD4^+^ T cells expressed higher levels of FOXP3 when cultured with anti-GM-CSF-treated monocytes in comparison with controls, and this effect was reversed by anti-CXCL-11 (Fig. 7C). In addition, the expression of RORγt which is related to Th17 cells was increased significantly after neutralization of CXCL-11 (Fig. 7D). Taken together, these data confirm that CXCL-11 is a major modulatory chemokine produced by anti-GM-CSF-treated monocytes.

## Discussion

The significant inflammatory role of GM-CSF in autoimmune disorders such as multiple sclerosis (MS) and rheumatoid arthritis (RA) has recently been shown^3,31^. Even though the mechanistic basis of the GM-CSF inflammatory role has not been fully elucidated, its biological and clinical implications are clear^32–36^. In this study, we show that CXCL-11 is a monocyte-derived modulatory chemokine that is suppressed explicitly by GM-CSF. We showed that GM-CSF-treated monocytes increased the expression of critical antigen-presenting molecules such as HLA-DR and CD86 as compared with the control group.

Consistent with our findings, previous studies have shown that GM-CSF primes macrophages for the production of pro-inflammatory cytokines such as TNF-α and IL-6 in response to LPS^37,38^. In addition, Croxford *et al*. have indicated that GM-CSF signaling induces an inflammatory signature in CCR2^+^Ly6C^hi^ monocytes and their progeny, which plays a critical role in tissue destruction^11^. In agreement with the known mechanism of action of GM-CSF, in our study, the increase in HLA-DR and co-stimulatory molecule expression in the presence of GM-CSF and its decrease after GM-CSF blockage suggest that the inflammatory impact of GM-CSF is mediated in part through class II molecules along with co-stimulatory markers^39^. Next, we investigated and provided evidence of the role of GM-CSF blocking on T cell responses. We hypothesized that CXCL-11 would be increased after GM-CSF blockade. We next asked whether the CXCL-11 level might affect immunomodulatory pathways of T cell responses, and we found that CXCL-11 suppressed T cell proliferation induced by GM-CSF-treated monocytes. Moreover, CXCL-11 inhibited GM-CSF-induced pro-inflammatory effects of monocytes on CD4^+^ T cells. On the other hand, we demonstrated that CXCL-11 is involved in suppression of T cell proliferation caused by GM-CSF and also that this chemokine is required for the immunomodulatory effects of monocytes on CD4^+^ T cells.

CXCL-11 is a chemokine that regulates cell trafficking via communication with a specific 7- transmembrane G protein-coupled receptor (GPCR)^40^. This chemokine plays a vital role in the induction of chemotaxis, tissue extravasation, and leukocyte differentiation^41^. Our findings of increased expression of PD-L1, CD39 (data not shown), and IL-10, with a decreased level of T cell proliferation and RORγt expression in treatments with rhCXCL-11 and anti-GM-CSF, show a critical relationship between GM-CSF and CXCL-11 in inflammation regulation. CXCL9 (MIG), CXCL10 (IP-10), and CXCL-11 (I-TAC) can bind to a chemokine receptor called CXCR3, which is expressed on effector T cells, Th17 and also NK cells^42–46^. The binding epitope site of CXCL-9 and CXCL-10 on CXCR3 is different from that of CXCL-11^45,46^. These three chemokines are largely secreted by monocytes, endothelial cells, fibroblasts, and cancer cells^47^. Notably, CXCL-11 has a much greater affinity for binding to CXCR3 than CXCL-9 and CXCL-10, resulting in desensitization of the receptor^45,46^, which makes it a possible antagonist of two other ligands.

In a mechanistic study, Paterka *et al*. assessed the role of CD11c^+^ cells in neuroinflammation. They indicated that IL-17 production by Th17 cells is induced by GM-CSF, and more interaction between Th17 cells and dendritic cells locally reactivates Th17 cells. This loop stimulates Th17 cells for the production of higher levels of GM-CSF, which is required for CD11c^+^ cell induction^48^. Furthermore, the role of GM-CSF in the induction of experimental autoimmune encephalomyelitis (EAE) is not related to IL-17 and IFN-γ^7,48^. CXCL-10 potentially drives Th1 cell differentiation, whereas it has been suggested that CXCL-11 induces FOXP3- negative regulatory T cells that suppress autoimmune encephalomyelitis^30,49^. The robust production of CXCL10 and CXCL9 by CD11c^+^ cells in EAE mice and the depletion of T cell retention or accumulation in the CNS of GM-CSF-deficient (Csf2^−/−^) and CD11c^+^- depleted mice also demonstrate the inflammatory effects of GM-CSF^48,50^. In addition, suppression of CXCL10 and CXCL-11-induced chemotaxis does not affect IL-10 and IFN-γ production by CXCL-11 and CXCL10, respectively^51^. Accordingly, it thus appears that CXCL-11 binding to CXCR3 regulates inflammatory immune responses that occur in the absence of GM-CSF^49^.

In summary, our findings indicate that GM-CSF blockade not only inhibited the pro- inflammatory profile of monocytes, but they also suggest an immunomodulatory role. Our study also strengthens the possibility that GM-CSF may contribute to inflammatory responses through suppression of CXCL-11 production. Induction of CXCL-11 after GM-CSF neutralization and enhanced production of immunoregulatory markers such as IL-10 and PD-L1 after CXCL-11 treatment indicate that CXCL-11 promotes anti-inflammatory responses and that GM-CSF likely interferes with that function. More studies are warranted to test if CXCL11 treatment can suppress chronic inflammatory autoimmune diseases, such as MS and RA.

## References

[1] Zhan Y, Xu Y, Lew AM (2012). The regulation of the development and function of dendritic cell subsets by GM-CSF: more than a hematopoietic growth factor. Mol. Immunol..

[2] Becher B, Spath S, Goverman J (2017). Cytokine networks in neuroinflammation. Nat. Rev. Immunol..

[3] Cook AD (2016). Granulocyte macrophage colony-stimulating factor receptor alpha expression and its targeting in antigen-induced arthritis and inflammation. Arthritis Res. Ther..

[4] Samarpita S, Doss HM, Ganesan R, Rasool M (2018). Interleukin 17 under hypoxia mimetic condition augments osteoclast mediated bone erosion and expression of HIF-1alpha and MMP-9. Cell Immunol..

[5] Hirota Keiji, Hashimoto Motomu, Ito Yoshinaga, Matsuura Mayumi, Ito Hiromu, Tanaka Masao, Watanabe Hitomi, Kondoh Gen, Tanaka Atsushi, Yasuda Keiko, Kopf Manfred, Potocnik Alexandre J., Stockinger Brigitta, Sakaguchi Noriko, Sakaguchi Shimon (2018). Autoimmune Th17 Cells Induced Synovial Stromal and Innate Lymphoid Cell Secretion of the Cytokine GM-CSF to Initiate and Augment Autoimmune Arthritis. Immunity.

[6] Makris A (2017). Increased Frequency of Peripheral B and T Cells Expressing Granulocyte Monocyte Colony-Stimulating Factor in Rheumatoid Arthritis Patients. Front. Immunol..

[7] El-Behi M (2011). The encephalitogenicity of T(H)17 cells is dependent on IL-1- and IL-23-induced production of the cytokine GM-CSF. Nat. Immunol..

[8] Hayashida K (1990). Molecular cloning of a second subunit of the receptor for human granulocyte-macrophage colony-stimulating factor (GM-CSF): reconstitution of a high-affinity GM-CSF receptor. Proc. Natl Acad. Sci. USA.

[9] Xu D (2015). Novel insights in preventing Gram-negative bacterial infection in cirrhotic patients: review on the effects of GM-CSF in maintaining homeostasis of the immune system. Hepatol. Int..

[10] Wicks IP, Roberts AW (2016). Targeting GM-CSF in inflammatory diseases. Nat. Rev. Rheumatol..

[11] Croxford AL (2015). The Cytokine GM-CSF Drives the Inflammatory Signature of CCR2+ Monocytes and Licenses Autoimmunity. Immun..

[12] Greter M (2012). GM-CSF controls nonlymphoid tissue dendritic cell homeostasis but is dispensable for the differentiation of inflammatory dendritic cells. Immun..

[13] Ko HJ (2014). GM-CSF-responsive monocyte-derived dendritic cells are pivotal in Th17 pathogenesis. J. Immunol..

[14] Vogel DY (2015). GM-CSF promotes migration of human monocytes across the blood brain barrier. Eur. J. Immunol..

[15] Wiggins-Dohlvik, K. *et al*. Tumor necrosis factor-alpha disruption of brain endothelial cell barrier is mediated through matrix metalloproteinase-9. *Am. J. Surg*. **208**, 954–960, discussion 960 (2014). 10.1016/j.amjsurg.2014.08.014 25312844

[16] Mahad DJ, Ransohoff RM (2003). The role of MCP-1 (CCL2) and CCR2 in multiple sclerosis and experimental autoimmune encephalomyelitis (EAE). Semin. Immunol..

[17] Sierra-Filardi E (2014). CCL2 shapes macrophage polarization by GM-CSF and M-CSF: identification of CCL2/CCR2-dependent gene expression profile. J. Immunol..

[18] Mahad D (2006). Modulating CCR2 and CCL2 at the blood-brain barrier: relevance for multiple sclerosis pathogenesis. Brain.

[19] Michlmayr D, McKimmie CS (2014). Role of CXCL10 in central nervous system inflammation. *International*. J. Interferon, Cytokine Mediator Res..

[20] Vazirinejad R, Ahmadi Z, Kazemi Arababadi M, Hassanshahi G, Kennedy D (2014). The biological functions, structure and sources of CXCL10 and its outstanding part in the pathophysiology of multiple sclerosis. Neuroimmunomodulation.

[21] Muller M, Carter S, Hofer MJ, Campbell IL (2010). Review: The chemokine receptor CXCR3 and its ligands CXCL9, CXCL10 and CXCL11 in neuroimmunity–a tale of conflict and conundrum. Neuropathol. Appl. Neurobiol..

[22] Liu MT, Keirstead HS, Lane TE (2001). Neutralization of the chemokine CXCL10 reduces inflammatory cell invasion and demyelination and improves neurological function in a viral model of multiple sclerosis. J. Immunol..

[23] Wu XB (2018). Spinal CXCL9 and CXCL11 are not involved in neuropathic pain despite an upregulation in the spinal cord following spinal nerve injury. Mol. Pain..

[24] Koper OM, Kaminska J, Sawicki K, Kemona H (2018). CXCL9, CXCL10, CXCL11, and their receptor (CXCR3) in neuroinflammation and neurodegeneration. Adv. Clin. Exp. Med..

[25] Cepok S (2009). Enhancement of chemokine expression by interferon beta therapy in patients with multiple sclerosis. Arch. Neurol..

[26] Couture A (2019). HLA-Class II Artificial Antigen Presenting Cells in CD4(+) T Cell-Based Immunotherapy. Front. Immunol..

[27] Mausberg AK, Jander S, Reichmann G (2009). Intracerebral granulocyte-macrophage colony-stimulating factor induces functionally competent dendritic cells in the mouse brain. Glia.

[28] Shiomi, A. & Usui, T. Pivotal roles of GM-CSF in autoimmunity and inflammation. *Mediators Inflamm* **2015**, 568543 (2015). 10.1155/2015/568543 PMC4370199 25838639

[29] Arasanz H (2017). PD1 signal transduction pathways in T cells. Oncotarget.

[30] Zohar Y (2014). CXCL11-dependent induction of FOXP3-negative regulatory T cells suppresses autoimmune encephalomyelitis. J. Clin. Invest..

[31] Pare A (2018). IL-1beta enables CNS access to CCR2(hi) monocytes and the generation of pathogenic cells through GM-CSF released by CNS endothelial cells. Proc. Natl Acad. Sci. USA.

[32] Ponomarev ED (2007). GM-CSF production by autoreactive T cells is required for the activation of microglial cells and the onset of experimental autoimmune encephalomyelitis. J. Immunol..

[33] Hirota K (2007). T cell self-reactivity forms a cytokine milieu for spontaneous development of IL-17+ Th Cell cause autoimmune arthritis. J. Exp. Med..

[34] Ganesan R, Rasool M (2017). Interleukin 17 regulates SHP-2 and IL-17RA/STAT-3 dependent Cyr61, IL-23 and GM-CSF expression and RANKL mediated osteoclastogenesis by fibroblast-like synoviocytes in rheumatoid arthritis. Mol. Immunol..

[35] Wright HL, Bucknall RC, Moots RJ, Edwards SW (2012). Analysis of SF and plasma cytokines provides insights into the mechanisms of inflammatory arthritis and may predict response to therapy. Rheumatol..

[36] Guo X (2018). Blockade of GM-CSF pathway induced sustained suppression of myeloid and T cell activities in rheumatoid arthritis. Rheumatol..

[37] Bergamini A (2000). Granulocyte-macrophage colony-stimulating factor regulates cytokine production in cultured macrophages through CD14-dependent and -independent mechanisms. Immunology.

[38] Kreutz M (1999). Granulocyte-macrophage colony-stimulating factor modulates lipopolysaccharide (LPS)-binding and LPS-response of human macrophages: inverse regulation of tumour necrosis factor-alpha and interleukin-10. Immunology.

[39] Hornell TM, Beresford GW, Bushey A, Boss JM, Mellins ED (2003). Regulation of the class II MHC pathway in primary human monocytes by granulocyte-macrophage colony-stimulating factor. J. Immunol..

[40] Proudfoot AE (2002). Chemokine receptors: multifaceted therapeutic targets. Nat. Rev. Immunol..

[41] Franciszkiewicz K, Boissonnas A, Boutet M, Combadiere C, Mami-Chouaib F (2012). Role of chemokines and chemokine receptors in shaping the effector phase of the antitumor immune response. Cancer Res..

[42] Qin S (1998). The chemokine receptors CXCR3 and CCR5 mark subsets of T cells associated with certain inflammatory reactions. J. Clin. Invest..

[43] Sallusto F, Lenig D, Mackay CR, Lanzavecchia A (1998). Flexible programs of chemokine receptor expression on human polarized T helper 1 and 2 lymphocytes. J. Exp. Med..

[44] Nakae S, Iwakura Y, Suto H, Galli SJ (2007). Phenotypic differences between Th1 and Th17 cells and negative regulation of Th1 cell differentiation by IL-17. J. Leukoc. Biol..

[45] Colvin RA, Campanella GS, Sun J, Luster AD (2004). Intracellular domains of CXCR3 that mediate CXCL9, CXCL10, and CXCL11 function. J. Biol. Chem..

[46] Colvin RA, Campanella GS, Manice LA, Luster AD (2006). CXCR3 requires tyrosine sulfation for ligand binding and a second extracellular loop arginine residue for ligand-induced chemotaxis. Mol. Cell Biol..

[47] Tokunaga R (2018). CXCL9, CXCL10, CXCL11/CXCR3 axis for immune activation - A target for novel cancer therapy. Cancer Treat. Rev..

[48] Paterka M (2016). Gatekeeper role of brain antigen-presenting CD11c+ cells in neuroinflammation. EMBO J..

[49] Karin N, Wildbaum G, Thelen M (2016). Biased signaling pathways via CXCR3 control the development and function of CD4+ T cell subsets. J. Leukoc. Biol..

[50] Codarri L (2011). RORgammat drives production of the cytokine GM-CSF in helper T cells, which is essential for the effector phase of autoimmune neuroinflammation. Nat. Immunol..

[51] Zohar Y (2017). CXCL11-dependent induction of FOXP3-negative regulatory T cells suppresses autoimmune encephalomyelitis. J. Clin. Invest..

